# The effect of multiple exposures in scenario‐based simulation—A mixed study systematic review

**DOI:** 10.1002/nop2.639

**Published:** 2020-09-29

**Authors:** Alette H. Svellingen, Margrethe B. Søvik, Kari Røykenes, Guttorm Brattebø

**Affiliations:** ^1^ Centre of Diaconia and Professional Practice VID Specialized University Oslo Norway; ^2^ Faculty of Health Studies VID Specialized University Bergen Norway; ^3^ Department of Anaesthesia & Intensive Care Haukeland University Hospital Bergen Norway; ^4^ Department of Clinical Medicine University of Bergen Bergen Norway

**Keywords:** clinical competence, education, learning, mixed study systematic review, multiple simulation, nursing students, scenario‐based simulation

## Abstract

**Aims:**

To examine the use and effects of multiple simulations in nursing education.

**Design:**

A mixed study systematic review. Databases (CINAHL, Medline, PubMed, EMBASE, ERIC, Education source and Science Direct) were searched for studies published until April 2020.

**Method:**

Researchers analysed the articles. Bias risk was evaluated using the Critical Appraisal Skills Programme and Cochrane Risk of Bias tool.

**Results:**

In total, 27 studies were included and four themes identified. Students participated in multiple simulation sessions, over weeks to years, which included 1–4 scenarios in various nursing contexts. Simulations were used to prepare for, or partly replace, students’ clinical practice. Learning was described in terms of knowledge, competence and confidence.

**Conclusion:**

Multiple scenario‐based simulation is a positive intervention that can be implemented in various courses during every academic year to promote nursing students’ learning. Further longitudinal research is required, including randomized studies, with transparency regarding study design and instruments.

## 1 INTRODUCTION

As they proceed through their professional training, nursing students face many challenges due to the complexity of health care and the numerous competence requirements. Before graduation, educational institutions must ensure that nursing students are able to care for real patients. The goal of nursing education is to support each student’s individual needs, ensuring their competence while simultaneously securing patient safety (McCaughey & Traynor, 2010).

Scenario‐based simulations have been widely incorporated into nursing education and are considered a valuable approach (Cant & Cooper, 2017a, 2017b). Such simulations can bring students’ theoretical and abstract understanding into a patient scenario (INACSL Standards Committee, 2016a). In this setting, students are active participants in the learning process and the debriefing should promote reflection and, ideally, reinforce learning (INACSL Standards Committee, 2016b). However, simulation programme development and usage are time‐consuming and costly (Lapkin & Levett‐Jones, 2011). To determine the amount of simulation training required and thus optimize investments in simulation, educators must know whether and how students achieve competence through simulation.

### 1.1 Background

Nursing student competence is a complex concept—combining knowledge, skills and performance. Moreover, the time available for developing competence in contact with real patients is increasingly limited. To help students transfer theoretical knowledge and alleviate “transition shock” (Beyea et al., 2010), simulation has become an important part of nursing education (Lavoie & Clarke, 2017).

In a recent review, Cerra et al. (2019) present strong evidence that high‐fidelity simulation (HFS) can improve learning compared with other teaching methods. Additionally, a systematic review shows that HFS positively contributes to students’ self‐confidence (Labrague et al., 2019). In a literature review, Kim and Yoo (2020) examined the use of debriefing in healthcare simulation and recommended that educators choose appropriate debriefing for learners to achieve maximum learning effects.

Clinical learning in real clinical settings allows nursing students to integrate theory with practice and maximize clinical competencies. Therefore, in all European Union countries, a nursing bachelor education must be completed at least half through supervised clinical practice (European Parliament. Directive, 2013/55). Simulations allow students to develop competence related to various medical fields—including paediatrics (Edwards et al., 2018), internal medicine and surgery (Kaddoura et al., 2016) and mental health (Olasoji et al., 2020). Simulation‐based learning can be combined with clinical practice to improve competence (Larue et al., 2015), but cannot yet fully substitute for supervised clinical practice.

To implement simulation as a substitute for direct experience with patients, it is important to determine the “dose” of simulation that best promotes learning. Therefore, it is essential to critically assess studies on the effects of multiple simulation sessions during an education programme. Earlier reviews have focused on simulation use, with the aim of identifying optimal strategies related to specific elements of the simulation session, but no reviews specifically summarize multiple simulation sessions. Thus, there is a need to analyse these studies to better understand of how several simulation sessions affects nursing students’ learning.

## 2 THE REVIEW

### 2.1 Aim

The specific aim of the review was to identify, describe and summarize evidence related to multiple simulation sessions in nursing education. This review was guided by two questions: “How are multiple simulations used as interventions to develop nursing students’ learning?” and “What is the effect of multiple simulations on nursing students’ learning?”

### 2.2 Design

For this mixed study review, we applied a convergent synthesis design (Hong et al., 2017; Pluye & Hong, 2014), for both qualitative and quantitative research. The results are reported according to the Preferred Reporting Items for Systematic Reviews and Meta‐Analyses (PRISMA) (Moher et al., 2009).

### 2.3 Search methods

The search strategy was based on an initial broad search developed by a research librarian in cooperation with the other authors. The search strategy included various terms relating to multiple simulations and education, with both medical subject headings and entry terms, as follows: “scenario‐based simulation” OR “clinical simulation” OR “simulation‐based learning” OR “simulation training” AND “simulation series” OR “simulation sessions” OR “multiple simulations” OR “repeated exposure” AND “nursing students” OR “nursing education.” Table S1 presents an example of a search string.

The study protocol was registered at PROSPERO (CRD42019117789, http://www.crd.york.ac.uk/PROSPERO/display_record.php?ID=CRD42019117789). The main searches were conducted in August 2018, with no imposed date range, in the following databases: CINAHL, Medline, PubMed, EMBASE, ERIC, Education Source and Science Direct. A follow‐up search was conducted to identify articles published between August 2018 and the end of April 2020. The results from the database searches were collected using the reference management software Zotero. After duplication control, the references were imported to the screening tool Rayyan. Reference lists from the included sources were checked to identify additional studies.

### 2.4 Search outcomes

**FIGURE 1:**
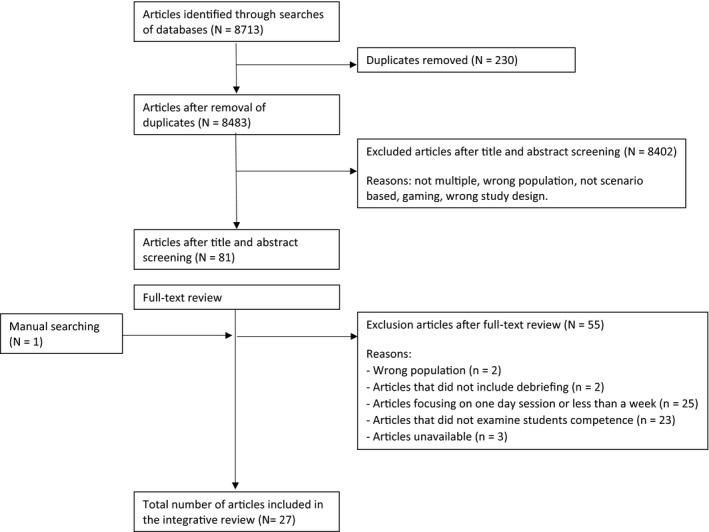
Flowchart

Broad inclusion criteria were applied to generate a comprehensive overview of multiple scenario‐based simulations at any stage during the nurse educational programme. We broadly defined multiple simulations as numerous scenario‐based simulation sessions, separated by over one week. A session comprises either one or several scenarios executed on the same day. Studies were not excluded based on language. Table 1 presents the inclusion and exclusion criteria. The first author screened 8,713 abstracts for relevance. The selected abstracts were then screened by three researchers against the inclusion criteria, reducing the number of articles to 81. Manual searching yielded the inclusion of one additional article. Thus, 82 articles were obtained in full text and each was reviewed by a minimum of two researchers. Finally, we included 27 articles. Figure 1 illustrates details of the selection process.

**TABLE 1:** Inclusion and exclusion criteria

Included	Excluded
Nursing students in all part of their educational program	Other healthcare students, nurses, medical staff or other professions
Articles include the influence of simulation dose on nursing students learning	Books and book chapters, conference proceedings, editorials
Scenario‐based simulation	Skills training
Debriefing as part of simulation	Evaluation of clinical interventions
Simulation in a period of time	One or two simulation sessions in less than a week
Qualitative, quantitative and mixed methods research	Theoretical articles
PhD dissertations	Computer‐based virtual simulation and gaming
Human patient actors or manikins	
Published in a peer‐reviewed journal	

### 2.5 Quality appraisal

To assess the risk of bias in the studies, the Critical Appraisal Skills Programme (CASP) was adapted to systematically appraise the methodological quality of the included articles (CASP, 2013). Three researchers rated the 27 studies using nine criteria: aim, design, methods, sample, ethical considerations, results, limitations, implications and study sponsor (Table S2). To supplement the CASP evaluation, the articles were also independently assessed for quality by pairs of researchers using the Cochrane Risk of Bias tool. This tool considers bias in terms of selection, performance, detection, attrition, reporting and other bias and rates studies as having a high, low or unclear risk of bias for each domain (Table S3). No studies were excluded due to inadequate rigour or substantial bias.

### 2.6 Data abstraction and synthesis

A convergent synthesis was adapted to incorporate the integration of qualitative and quantitative data into the results (Pluye & Hong, 2014). Articles were analysed by three researchers—first individually and then the researchers discussed and identified topics for thematic analysis. In the first stage, results were extracted from all studies and entered into a table to compare characteristics and main results. Next, the data were compared to identify patterns and themes. Finally, the results extracted from all articles were categorized and thematically coded for similarities. Qualitative studies were read several times to identify concepts linking them to the research questions.

## 3 RESULTS

Four themes emerged that addressed the two questions guiding this review: time frame, context and number of scenarios in each simulation session, multiple simulations in clinical placement and effect on students’ learning.

### 3.1 Characteristics of the studies

Table 2 presents characteristics of the included studies. The studies originated from eight countries: 18 from the USA, 2 each from the UK and South Korea and 1 each from Spain, Australia, Hong Kong, Oman and Singapore. Among the studies, 2 were qualitative, 24 quantitative and 1 mixed methods. The used data collection methods included video‐recorded observations (*N = *1), surveys (*N = *13), evaluation instruments (*N = *10) and focus group interviews (*N = *2), either alone or in combinations. The studies included three dissertations for the degree of Doctor of Nursing.

**TABLE 2:** Summary of included studies

	Authors, year and location	Study aim	Simulation setting	Simulation intervention	Design	Sample*	Key findings
1	Bussard (2018)**USA**	examine progression in clinical judgement	Medical‐surgical nursing course. HFS	One session every third to fourth week throughout the semester Group size: unknown	Quantitative descriptive study design	*n* = 70 junior students	Results showed a progression of clinical judgement between the first and final simulation session
2	Chiang and Chan (2014)**Hong Kong**	evaluate the development of critical thinking disposition	Adult nursing courses. HFS	Two sessions over two semester Group size: 3–4	Mixed‐method. Pre/post questionnaires and 4 focus group interviews	*n* = 71 second year students	Critical thinking disposition scores increased significantly after two semesters. Qualitative data did not focus on multiple simulation
3	Cummings and Connelly (2016)**USA**	identify students confidence level with repeated simulation activities	Adult health I course, adult health II course.	Six scenarios over a one year period Group size: 3–4	Quantitative study design. Survey	*n* = 54 students 34 junior students. 20 senior students	Repeated simulation activities can increase student confidence levels
4	Curl et al. (2016)**USA**	investigate the impact of replacing 50% of traditional clinical practice with simulation	Four clinical specialty; obstetric, paediatric, mental health and critical care. HFS	20 sessions over the first semester Group size: 5	Quantitative, quasi‐experimental. Pre/post questionnaire	*n* = 97 students *n* = 50 experimental group *n* = 47 control group	Substituting half of traditional clinical practice resulted in significantly higher scores than traditional clinical experiences alone
5	Díaz Agea et al. (2018)**Spain**	analyze perceptions of the process of learning ethics and describe frames that precede ethical actions	Simulation of ethical content HFS	Two sessions, separated by 1 week Group size: 10	Qualitative study, analyzing video‐recorded simulation	*n* = 30 fourth year students	Students were satisfied with the opportunity to repeat the simulation experience
6	Hansen and Bratt (2017)**USA**	explore the effect of sequence of simulated and clinical practicum learning experiences on clinical competence	Medical‐surgical nursing practicum course HFS	Three sessions over a 7 week period Group size: 7–8	Quantitative, randomized crossover design	*n* = 48	Findings revealed no differences in competency scores between the two groups
7	Hart et al. (2014)**USA**	evaluate students’ performance in recognizing and responding to deteriorating patients	Acute Patient Deterioration course (45 h). HFS	Three sessions during a course Group size: 4–5	Quantitative, quasi‐experimental repeated measures design	*n* = 48	Significant increases were shown in time to emergency response and performance in recognizing and responding to APD events
8	Hicks et al. (2009)**USA**	compare the effectiveness of simulation to actual clinical experience among nursing students	Critical care course. HFS	30‐hour simulation experience Group size: unknown	Quantitative, randomized pre/post‐test design	*n* = 58 Senior students Simulation group Combo group Clinical group	The overall knowledge and performance score between groups were not statistically sign, students in the combo and clinical groups were consistently rated higher on performance score than students in the simulation groupStudents in simulation and combo groups had a statistically significant increase in self‐confidence level
9	Hill (2014)**USA**	identify if multiple exposures can improve students’ clinical performance	Chronic obstructive pulmonary disease scenario. HFS	Three sessions throughout an academic year Group size: 3–5	Quantitative, repeated measures design	*n* = 52 *n* = 9 campus A *n* = 18 campus B *n* = 27 campus C	The results indicate a significant growth in knowledge from the first simulated experience to the last
10	Hoffmann et al. (2007)**USA**	investigate if HFS in conjunction with a traditional clinical experience improves basic knowledge of critical care nursing	Medical‐surgical nursing course. HFS	A weekly session for 7 weeks Group size: 7–8	Quantitative, repeated‐measure design	*n* = 29 Senior students	Results showed a significant improvement on basic knowledge of critical care nursing
11	Ironside et al. (2009)**USA**	Investigate if experiences with multiple‐patient simulation improve students’ patient safety competencies	Management Course ‐ acute care nursing. HFS	Two session over a 6‐week period Group size: 5	Quantitative, pre/post‐test design	*n* = 67 Senior students	There were significant differences in patient safety competencies from the first to final session
12	Lacue (2017) **USA**	determine the effect on students who experience a repeated scenario design of simulation to their overall self‐confidence and performance	Medical‐surgical nursing course. HFS	Two simulation days, 3 weeks apart Group size: 4	Quantitative, non‐experimental design	*n* = 24 Junior students	The overall competence score showed a statistically significant change from the first simulated experience to the last. Results did not indicated that student self‐confidence was positively impacted with a repeated simulation design
13	Liaw et al. (2014)**Singapore**	evaluate the outcomes of a simulation program preparing students for their transition to graduate nurse practice	Transitional‐to‐practice course. HFS	A weekly session for 5 weeks Group size: 6	Quantitative, quasi‐experimental pre/post‐test design	*n* = 91 Senior students	Post test scores indicated that the students perceived higher level of preparedness for their transition to graduate nurse clinical practice
14	Mancini et al. (2019)**USA**	understand the role that simulation and traditional clinical instruction plays on clinical competence	Four clinical specialty; medical‐surgical, paediatric, obstetric and critical care. HFS	Four sessions over four semester Group size: unknown	Quantitative, experimental, multisite study	*n* = 586 Junior and senior students *n* = 315 experimental group *n* = 271 control group	There were no significant differences on examination pass rates nor in clinical competency between students who participated in simulation and those combining simulation and clinical experience
15	Melenovich (2012)**USA**	explore the impact of additional simulation experiences on the acquisition of critical thinking skills	Medical‐surgical nursing. HFS	Five versus three sessions over a 3 week period Group size: unknown	Quantitative, randomized, experimental, pre/post‐test design	*n* = 73 First semester students *n* = 36 experimental group *n* = 37 control group	Results indicated while not statistically significant, that the students in experimental group perceived higher mean score differences from pretest to post‐test when compared to the control group
16	Meyer et al. (2011)**USA**	evaluate the effects of a paediatric simulation curriculum on nursing students’ clinical performance	Paediatric course. HFS	Four simulations days, over a 2‐week period Group size: 5	Quantitative randomized repeated‐measure design	*n* = 116 Junior students	The results demonstrated improvement in clinical performance, but no significant results.
17	Mould et al. (2011)**Australia**	determine the feasibility and acceptability of the simulation series	Critical care scenarios. HFS	Three sessions per week over a 9‐week period Group size: 4	Quantitative Pre/post‐test design	*n* = 219 Senior student	Both competence and confidence score improved significantly over time
18	Moule et al. (2008)**United Kingdom**	investigate student knowledge in manual handling and basic life support	Different medical scenarios	Five days of simulation, weeks unknown Group size: unknown	Mix method Pre/post‐test design	*n* = 62 First and third year students	Students’ knowledge increased between the pre and post scores, though not significantly
19	Najjar et al. (2015)**USA**	examine the experiences of nursing students in high‐fidelity simulation	Different baccalaureate nursing programs. HFS	Between four and twelve sessions per academic year Group size: 8–30	Qualitative design. Focus group interviews	*n* = 26	Students became more comfortable in simulation sessions during the years and were able to better prepare for and process through simulation sessions
20	Raman et al. (2019)**Oman**	compare the effects of a combination of traditional clinical training with HFS vs traditional clinical training alone on the clinical competency and knowledge	A maternity nursing course. HFS	34‐hour simulation (25% of the clinical hours) Group size: unknown	Quantitative, quasi‐experimental design	*n* = 74 Level 4 under‐graduate students *n* = 34 experimental group *n* = 40 control group	The results demonstrated no significant differences on clinical competency between groups
21	Roh et al. (2020)**South Korea**	To identify the effects of simulation with team‐based learning on knowledge, team performance and teamwork	Adult health nursing scenarios in a team‐based course. HFS	Four sessions over a 15 week period Group size: 4	Quantitative Pre/post‐test design	*n* = 229 fourth year students	Statistically significant higher knowledge score in the Group Readiness Assurance Test than in the Individual Readiness Assurance Test. Statistically significant post‐test scores on team performance and teamwork.
22	Schlairet and Pollock (2010)**USA**	explore student knowledge acquisition	A fundamental nursing course. HFS	2 weeks of simulation, sessions unknown Group size: unknown	Quantitative, randomized crossover design	*n* = 71 undergraduate students	Knowledge scores were statistically equivalent for both simulated and traditional clinical experiences
23	Schlairet and Fenster (2012)**USA**	identify a model to promote development of clinical judgement	Basic nursing concepts and skills course. HFS	Various dose of simulation during 6 weeks; Two days, three days or four days Group size: 4–5	Mixed‐methods Pre/post‐test design	*n* = 78 Junior students	No difference in critical thinking post‐test scores or the final examination score were observed by design. Clinical judgement scores were positive with the 50% simulation design.
24	Shin el al. (2015)**South‐Korea**	identify the effects of differing numbers of simulation exposures on critical thinking skills	A paediatric nursing clinical course. HFS	Various dose of simulation during 3 weeks; one, two or three sessions Group size: 2–3	Quantitative Pre/post‐test design.	*n* = 237 Senior students *n* = 110 cohort A *n* = 54 cohort B *n* = 73 cohort C	Critical thinking scores varied according to number of exposures. Three simulation sessions gained significant scores
25	Thomas and Mackey (2012)**USA**	examine the effects of high‐fidelity simulation on students’ level of confidence	A clinical simulation course. HFS	One session per week throughout the semester Group size: 5–7	Quantitative quasi‐experimental Pre/post‐test design	*n* = 24 *n* = 14 experimental group *n* = 10 control group	Students attending simulation were significantly more confident in all four dimensions (Recognition, assessment, intervention and evaluation) compared with students in a traditional clinical course
26	Unsworth et al. (2016) **United Kingdom**	explore discrepancy between students’ current and perceived performance	Medical scenarios, related to recognition and rescue of the deteriorating patient. HFS	Three sessions throughout an academic year Group size: 4–6	Quantitative, quasi‐experimental case study design	*n* = 70 Second students	Results report significant difference in performance between the first and the final scenario
27	Zapko et al. (2018)**USA**	examine student perception of best educational practices in simulation and evaluated student satisfaction and self‐confidence	Simulation scenarios related to different courses. HFS	Two sessions throughout two years Group size: unknown	Quantitative descriptive study design	*n* = 199 different level of students	Students responded positively to serial simulations related to both learning and self‐confidence. It was a significant difference between Year 1 and Year 2 in terms of high expectations, the importance of collaboration, diverse learning and the high expectations

* The definitions of student level vary between the articles, (e.g., senior and third year students) and are presented as described.

The time frame over which the studies were conducted varied between two weeks, several semesters and years. Simulation sessions were related to different medical courses, including paediatric, medical–surgical, mental health, critical care, acute patient deterioration and adult nursing courses. Additionally, one study focused on simulated scenarios with ethical content and another implemented simulation in a transition‐to‐practice course.

Among the quantitative studies, six used a randomized study design (Hansen & Bratt, 2017; Hicks et al., 2009; Melenovich, 2012; Meyer et al., 2011; Schlairet & Fenster, 2012; Schlairet & Pollock, 2010). In 10 studies, data were collected using a scoring sheet to evaluate students’ competence (Bussard, 2018; Chiang & Chan, 2014; Curl et al., 2016; Hart et al., 2014; Hill, 2014; Ironside et al., 2009; Lacue, 2017; Mancini et al., 2019; Raman et al., 2019; Shin et al., 2015) and 13 studies employed self‐report surveys (Chiang & Chan, 2014; Cummings & Connelly, 2016; Hoffmann et al., 2007; Ironside et al., 2009; Lacue, 2017; Liaw et al., 2014; Mould et al., 2011; Moule et al., 2008; Raman et al., 2019; Roh et al., 2020; Thomas & Mackey, 2012; Unsworth et al., 2016; Zapko et al., 2018).

### 3.2 Time frame

Table 3 presents the variations in time frame. Most studies examined students’ participation in simulations over a period of one semester or less. Seven studies described simulations over a time frame of two to five weeks. Díaz Agea et al. (2018) videotaped students participating in two sessions held one week apart. In another study, students attended four simulations over a two‐week period (Meyer et al., 2011). Lacue (2017) implemented two simulation days held three weeks apart, to determine the effects on students’ overall self‐confidence and clinical skill performance. Two studies held weekly simulation sessions—Melenovich (2012) over three weeks and Liaw et al. (2014) for five weeks. Schlairet and Pollock (2010) implemented simulation experiences for two weeks. In a multi‐site study, Shin et al. (2015) identified the effects of differing numbers of exposures, letting students participate in simulations for one, two or three weeks.

**TABLE 3:** Time frame

2–9 weeks	One semester	One year	Two years
Díaz Agea et al. (2018)	Bussard (2018)	Chiang and Chan (2014)	Zapko et al. (2018)
Hansen and Bratt (2017)	Curl et al. (2016)	Cummings and Connelly (2016)	Mancini et al. (2019)
Hoffmann et al. (2007)	Thomas and Mackey (2012)	Hill (2014)	
Ironside et al. (2009)	Roh et al. (2020)	Najjar et al. (2015)	
Lacue (2017)		Unsworth et al. (2016)	
Liaw et al. (2014)	**Period of simulation referred by hours**		
Melenovich (2012)	Hicks et al. (2009)		
Meyer et al. (2011)	Hart et al. (2014)		
Mould et al. (2011)	Moule et al. (2008)		
Schlairet and Pollock (2010)	Raman et al. (2019)		
Schlairet and Fenster (2012)			
Shin el al. (2015)			

Note

Articles sorted by period of simulation.

Five studies described simulations over a period of six to nine weeks. Mould et al. (2011) reported simulation sessions over nine weeks, where each student had hands‐on simulations in 18 scenarios and observer roles in nine other scenarios. Hoffmann et al. (2007) examined students participating in weekly sessions for seven weeks. Hansen and Bratt (2017) performed a randomized crossover study, implementing three days of simulation over seven weeks. In the study of Schlairet and Fenster (2012), first‐semester students experienced simulations with varying doses over six weeks. One study implemented multiple‐patient simulations during two sessions, separated by five weeks (Ironside et al., 2009).

To identify the effects of simulation, Roh et al. (2020) implemented sessions over a 15‐week period. In another study, students attended one simulation session every week during a semester (Thomas & Mackey, 2012). Five studies reported a series of simulations throughout an academic year (Chiang & Chan, 2014; Cummings & Connelly, 2016; Hill, 2014; Najjar et al., 2015; Unsworth et al., 2016). Two studies examined simulation sessions over a two‐year period: one where students participated in two‐day simulation sessions (Zapko et al., 2018) and another where four simulation sessions were implemented throughout fours semesters (Mancini et al., 2019).

Four studies did not specify the time period, but referred to 30 hr (Hicks et al., 2009), 34 hr (Raman et al., 2019), 45 hr (Hart et al., 2014) and five days (Moule et al., 2008) of scenario‐based simulation.

### 3.3 Context and number of scenarios in each simulation session

Students participated in several simulation sessions during the educational programmes, with varying numbers of scenarios in each session. Table 4 shows that simulation sessions varied between one–four scenarios, implemented in different contexts. Seven studies described multiple sessions over a time period, without clearly defining the number of scenarios in each session (Cummings & Connelly, 2016; Hicks et al., 2009; Hoffmann et al., 2007; Mancini et al., 2019; Moule et al., 2008; Najjar et al., 2015; Schlairet & Fenster, 2012; Schlairet & Pollock, 2010). In 25 studies, HFS sessions were implemented and only two studies did not specify fidelity level (Cummings & Connelly, 2016; Moule et al., 2008).

**TABLE 4:** Context and number of scenarios in each simulation session

No. of scenarios per session	Study	Context
1 scenario per session	Bussard (2018)	Medical‐surgical nursing course
	Hart et al. (2014)	Acute Patient Deterioration course
	Hill (2014)	Chronic obstructive pulmonary disease
	Ironside et al. (2009)	Acute care nursing course
	Raman et al. (2019)	Maternity nursing course
	Roh et al. (2020)	Adult health nursing scenarios
	Thomas and Mackey (2012)	High fidelity clinical simulation course
2 scenarios per session	Chiang and Chan (2014)	Adult nursing course
	Lacue (2017)	Medical‐surgical nursing course
	Unsworth et al. (2016)	The deteriorating patient
	Curl et al. (2016)	Four clinical specialty, not specified
3 scenarios per session	Mould et al. (2011)	Critical care scenarios
	Shin et al. (2015)	Paediatric course
	Díaz Agea et al. (2018)	Ethical course
	Melenovich (2012)	Medical‐surgical nursing course
4 scenarios per session	Meyer et al. (2011)	Paediatric course
	Hansen and Bratt (2017)	Medical‐surgical nursing scenarios
	Liaw et al. (2014)	The transition‐to‐practice course
	Zapko et al. (2018)	Different combinations; basic nursing scenarios, paediatric, medical/surgical, mental health, geriatric and community scenarios

### 3.4 Contributions of multiple simulation to prepare and substitute for clinical placements

In most studies evaluating multiple simulations over a semester or more, sessions were not held in conjunction with clinical practice. Curl et al. (2016) and Mancini et al. (2019) each implemented simulation across four clinical specialties during a semester, to better understand the impact of replacing traditional practice with simulation. Ten other studies evaluated multiple simulations related to students’ clinical practice, but each over less than a semester.

Liaw et al. (2014) describe the contribution of multiple simulations in preparation for clinical placement, reporting a significantly increased overall Preparedness Score from pre‐test to post‐test. Students’ written comments revealed that the simulation programme was helpful for understanding a nurse’s role. Two studies investigated two different sequences of blocks of simulated and traditional clinical experience. Schlairet and Pollock (2010) identified knowledge gains related to multiple sessions, which were as robust as gains related to traditional clinical placements. Students attended the simulated or traditional clinical experience and then changed to the opposite intervention after two weeks. Hanson and Bratt (2017) studied students who participated for seven weeks in the simulation laboratory and then switched roles with students in a medical–surgical practicum. They found that the students’ competency was not significantly associated with the sequence to which they were assigned.

Seven studies examined the use of multiple sessions as substitutes for traditional clinical placements. In a paediatric course (Meyer et al., 2011), students attended 25% of their clinical practicum in simulation. Throughout an eight‐week clinical rotation, students’ performance was evaluated every second week. Students who first participated in the simulation had higher performance scores than those who participated later in the course, although this difference was non‐significant. This study indicated that early exposure to simulation allowed students to more quickly achieve competence in the clinical unit. Similarly, Hoffmann et al. (2007 examined senior students’ knowledge and reported evidence supporting the efficacy of simulation in conjunction with traditional clinical experience. In this study, 50% of the clinical practicum was substituted with simulation. In an experimental study by Raman et al. (2019), students were exposed to simulation for 34 hr (25% of clinical hours) during their clinical rotation. The groups did not significantly differ in knowledge or clinical competency scores. Schlairet and Fenster (2012) examined simulation dose and sequence. In the open question part of the survey, students expressed that this type of learning helped them adapt to different clinical experiences, although the results were non‐significant.

The studies of Hicks et al. (2009) and Shin et al. (2015) each had three cohorts of students experiencing different simulation doses. Hicks et al. (2009) randomized students to one of three interventions: simulation only, combination of clinical and simulation or clinical experience only. The groups did not significantly differ in overall performance. Shin et al. (2015) reported that a cohort that attended a single simulation session had no significant gains in critical thinking, whereas students who had three simulation exposures attained significant gains.

In a British study (Moule et al., 2008), students missed six days from their clinical practicum to complete simulated scenarios on various topics. Significant results support the development of knowledge and skills in a range of clinical practice scenarios from simulations.

### 3.5 Effect on students’ learning

The final theme is the effect of multiple simulations on students’ learning. The core outcomes are represented in three sub‐categories: knowledge, competence and confidence. The studies used different instruments to analyse students’ learning.

#### 3.5.1 Knowledge

Five studies reported that multiple simulations benefited students in terms of knowledge acquisition (Curl et al., 2016; Hoffmann et al., 2007; Melenovich, 2012; Moule et al., 2008; Schlairet & Pollock, 2010). However, only two were randomized studies (Melenovich, 2012; Schlairet & Pollock, 2010).

Studies have revealed that multiple simulations appeared to have significant impact on students’ knowledge, using a HESI medical–surgical specialty examination (Curl et al., 2016) and the Basic Knowledge Assessment Tool‐6 (Hoffmann et al., 2007). Another study (Schlairet & Pollock, 2010) used a multiple‐choice questionnaire (MCQ) and revealed a significant increase in knowledge, although it was statistically equivalent between students in simulated and traditional clinical experiences. In two other studies, students somewhat increased their knowledge score, but the change was not significant, based on a HESI knowledge examination (Melenovich, 2012) and a MCQ of knowledge in practical handling and basic life support (Moule et al., 2008). Hicks et al. (2009) evaluated scores from existing examinations for the course and found no between‐group differences in knowledge.

#### 3.5.2 Competence

Different terms were used to describe students’ clinical competence: clinical judgement, critical thinking, clinical and patient safety competence and performance. Bussard (2018) and Schlairet and Fenster (2012) reported progression of clinical judgement between the first and final simulation sessions using the Lasater Clinical Judgment Rubric (LCJR). Schlairet and Fenster’s (2012) randomized 78 junior students to various simulation doses and found no significant change in clinical judgement.

Four studies focused on students’ critical thinking. Only one study (Chiang & Chan, 2014) found that multiple simulation sessions yielded significant improvement in overall critical thinking, using the California Critical Thinking Disposition Inventor (CCTDI). Melenovich (2012) used the same instrument and compared five versus three simulation sessions over three weeks, among 72 randomized students. The experimental group (five sessions) showed higher mean scores, though this difference was not significant. In a non‐randomized multi‐site study, Shin et al. (2015) used Yoon’s Critical Thinking Disposition Tool and revealed no significant differences in overall critical thinking scores between three cohorts. In addition to evaluating clinical judgement in their randomized study, Schlairet and Fenster (2012) assessed students’ critical thinking skills and showed no difference in post‐test scores according to simulation design.

Six studies evaluated the effect of multiple simulations on students’ clinical and patient safety competence. In three studies, competence scores significantly increased between the first and the final scenario. Students’ learning was evaluated using Quality and Safety in Education in Nursing (Ironside et al., 2009), Creighton Competency Evaluation Instrument (CCEI) (Lacue, 2017) and a survey developed specifically for one study (Mould et al., 2011). Hansen and Bratt (2017), Mancini et al. (2019) and Raman et al. (2019) also used CCEI, exploring the effect of the sequence of simulations and clinical practice and finding no between‐group differences in clinical competence scores.

Performance is the final concept presented to determine students’ competence. One study (Hart et al., 2014) used the Emergency Response Performance Tool and Patient Outcome Tool to evaluate students’ performance in recognizing and responding to deteriorating patients. Over three sessions, students showed significant increases in performance and time to emergency response. Over a one‐year period, Unsworth et al. (2016) implemented three simulation sessions related to recognition and rescue of the deteriorating patient. A “Discrepancy Discovery data collection tool” was developed to allow students to select aspects of their performance to develop before the next simulation session. The results showed significant differences in performance from the first to the last simulation experience. In a multi‐site study (Hill, 2014), students were exposed to a scenario three times throughout an academic year. Data were collected using the Creighton Simulation Evaluation Instrument tool and revealed significantly improved performance from the first to the third exposure. In another study, Hicks et al. (2009) used a specifically developed performance evaluation tool and found no significant effect on students’ performance scores. Meyer et al. (2011) also found no significant results when evaluating student performance using a tool adapted from the Likert‐style tool used by Massey and Warblow’s (2005). Roh et al. (2020) used a 20‐item checklist and reported significantly improved post‐test scores on team performance and teamwork.

In a qualitative study, Díaz Agea et al. (2018) analysed video‐recorded simulations and reported positive learning from attending two sessions relating to ethical competence. The authors mentioned that repeating the experience was an important factor for learning.

#### 3.5.3 Confidence

Among eight studies examining students’ confidence, six showed that significant improvement of confidence score over time. Hicks et al. (2009) developed the Self*‐*Confidence Scale for use in analysing how simulation may influence students’ confidence levels compared with clinical experience. Four dimensions describe students’ ability to recognize, assess, intervene and assess the effectiveness of implemented interventions—all in the respiratory, cardiac and neurological areas. The results indicated significantly increased self‐confidence among students with simulation experiences or with combined simulation and traditional clinical experiences, but not among students who only participated in clinical experience. Thomas and Mackey (2012) used the same instrument and reported significant between‐group differences in all four dimensions.

Studies with a quasi‐experimental design also revealed significantly increased confidence scores. In a study involving two simulation days, three weeks apart, the confidence score significantly improved over time (Lacue, 2017). Zapko et al. (2018) reported that multiple simulation experiences over a two‐year period seem to increase students’ confidence. Australian researchers (Mould et al., 2011) developed a questionnaire for their study and found significantly increased self‐confidence scores after simulation over nine weeks. Liaw et al. (2014) used the Preparedness for Hospital Practice Questionnaire and reported significantly improved confidence levels after a simulation programme preparing students for their transition to graduate nursing. Cummings and Connelly (2016) used “The Self‐Confidence in Learning” and found that repeated simulation can increase student confidence levels, although the results are poorly presented, making it difficult to draw conclusions.

Results from focus group interviews with students indicated confidence development during years of simulation sessions. Students became more comfortable and emphasized that multiple simulations enabled them to predict what would happen during sessions and that simulation was a safe arena where to learn from mistakes (Najjar et al., 2015).

### 3.6 Risk of bias

Most studies showed moderate methodological quality. Based on CASP and the Cochrane Risk of Bias assessment (Table S3), the major risk of bias in the experimental studies was due to lack of participant blinding, which is difficult to achieve in educational interventions. In the quasi‐experimental studies, the bias risk was related to non‐random sampling. Most studies had a low reporting bias and clearly presented the findings. Studies also reported whether participants had withdrawn.

## 4 DISCUSSION

This review aimed to explore how multiple simulations are used in nursing education and describe the effects of multiple exposures to scenario‐based simulation. The findings demonstrated that educators use multiple simulations in a range of nursing courses. Analysis of the studies showed that multiple simulations can be used in different ways, in terms of number of sessions and number of scenarios in a session.

Most studies used a pre‐ and post‐test design, with surveys and objective evaluations. These studies demonstrated positive results, supporting the use of multiple simulations in nursing education. Ten studies had sample sizes of <60 participants. More robust sample sizes would have increased the generalizability. Most studies reported significant findings, with participant numbers varying from 24 (Lacue, 2017; Thomas & Mackey, 2012)–586 (Mancini et al., 2019). Significant results were obtained both in sessions over two weeks (Schlairet & Pollock, 2010) and two years (Zapko et al., 2018). The use of a randomized study design (Hansen & Bratt, 2017; Hicks et al., 2009; Melenovich, 2012; Meyer et al., 2011; Schlairet & Fenster, 2012; Schlairet & Pollock, 2010) increased the validity, despite small sample sizes.

The use of multiple simulations has been widely studied; however, there is no clear relationship between students’ outcome and simulation dosage in terms of time frame or number of sessions and scenarios. Hoffmann et al. (2007) implemented a total of four scenarios over seven weeks, but with only 29 participants. Mould et al. (2011) examined 219 students participating in 18 scenarios over nine weeks. These two studies differed by only two weeks, but by 12 scenarios. Zapko et al. (2018) investigated students attending simulations over two years, with a total of eight scenarios.

Both Ironside et al. (2009) and Mould et al. (2011) reported significantly increased competence scores over time. Notably, these studies used different instruments. Moreover, while Ironside et al. (2009) were transparent regarding outcome measurement, Mould et al. (2011) did not specify the type of questions used.

Nurse educators strive to foster the necessary competence for students’ future clinical practice. In a review, Jeppesen et al. (2017) refer to several strategies to increase learning, indicating that simulation motivates students to learn. In general, repetitive practice will improve performance. However, designing and implementing multiple simulations require time and resources (Lin et al., 2018; Maloney & Haines, 2016). Providing simulations for large student cohorts can be challenging. Implementing simulation as part of students’ clinical practice enables organization of this intervention during a nursing course. Our present findings suggest that combining multiple simulation sessions with clinical placement decreases the gap between theory and practice. Debates currently surround the difficulties of transitioning to professional practice, both from the bachelor programme (Strickland & Welch, 2019) and for new graduate nurses (Chen et al., 2017).

Although multiple simulations influence students’ knowledge and competence, we found that only two studies presented significant results regarding knowledge score (Curl et al., 2016; Hoffmann et al., 2007). Effects on students’ learning outcomes can span a wide range, as competence is characterized by clinical judgement (Bussard, 2018; Schlairet & Fenster, 2012), critical thinking (Chin et al., 2014; Melenovich, 2012; Schlairet & Fenster, 2012; Shin et al., 2015), clinical competency (Hansen & Bratt, 2017; Ironside et al., 2009; Lacue, 2017; Mancini et al., 2019; Mould et al., 2011; Raman et al., 2019) and performance (Hart et al., 2014; Hicks et al., 2009; Hill, 2014; Meyer et al., 2011; Roh et al., 2020; Unsworth et al., 2016). Four studies reported significant between‐group differences (Chiang & Chan, 2014; Ironside et al., 2009; Lacue, 2017; Mould et al., 2011). Some studies reported no significant differences between groups exposed to multiple simulation as a substitute for clinical placements (Mancini et al., 2019; Raman et al., 2019). These studies bolster the view that simulations support students’ learning.

Students in this review generally reported high levels of learning in relation to multiple simulations. However, another review (Cantrell et al., 2017) revealed that simulation affects students’ emotions and increases their stress and anxiety levels. Najjar et al. (2015) reported that increased confidence over time improved the students’ ability to prepare for and make progress in simulation sessions. Self‐confidence is a foundation for learning (Woda et al., 2017), and our present findings show that participating in several simulation sessions can increase students’ self‐confidence, although only two studies had an experimental design (Hicks et al., 2009; Thomas & Mackey, 2012). Increased confidence was related to the decision‐making process (Hicks at al., 2009; Thomas & Mackey, 2012), confidence in learning with simulation (Lacue, 2017; Zapko et al., 2018) and coping with emotions and stress in the simulation session (Liaw et al., 2014). These results show that confidence is widely described and not necessarily transferable between sessions.

In non‐randomized studies, students seemed to attain greater experience, competence and confidence. Such studies were rated as having a low to high risk of bias (Table S2). Twelve studies in this review lacked a control group, which decreases the validity. All of them used pre‐test and post‐test designs to examine progression in student learning. One non‐randomized study (Shin et al., 2015) included 237 senior students from three schools—a sample size that increases the validity. Another quantitative study (Ironside et al., 2009) lacked an experimental design; however, all 69 students underwent the same intervention of multiple simulations and were evaluated on patient safety competencies. Thomas and Mackey (2012) performed a study of 24 students with a quasi‐experimental design. The results carry a high risk of bias, as there were only 14 students in the experimental group and 10 in the control group.

Research evaluating the effects of simulation must apply valid and reliable instruments, as this can influence the results and generalizability of findings. The studies in this review employed a mix of well‐validated and less well‐validated instruments, and most authors provided some discussion of the reliability and validity of the instruments used. The nursing examinations are considered valid and reliable standardized assessments of students’ knowledge. Ten studies described the validity of the instruments used to measure competence. CCEI, LCJR and CCTDI are well‐known and validated instruments for measuring the effectiveness of clinical learning through simulation. Additionally, four instruments were developed specifically for the studies and their validity was not specified. Instruments measuring confidence all involved students self‐reporting their reactions to the simulation. Reliability was tested using Cronbach’s alpha, which was between 0.87–0.97. One study developed an instrument specifically for measuring confidence in that study and did not specify the reliability.

### 4.1 Limitations

This review did not include studies of other healthcare students, thus limiting the generalizability of findings to other student categories. Additionally, some of the included studies did not clearly report all relevant aspects of their methods, context and findings (Table S2), making it difficult to assess the results.

## 5 CONCLUSION

The present review provides support for using multiple simulations. However, it offers no clear answer to the questions regarding the minimal effective simulation dose, that is how many scenarios or sessions should be implemented to maximize students’ learning. It appears to be beneficial to combine simulation and clinical placement. Little is known about how multiple simulations experienced over more than a year has an impact on students’ learning, and there are few randomized studies. In educational research, both randomized and longitudinal studies can be challenging due to the complexity of educational programmes. Further research should implement simulations in a longitudinal perspective and provide detailed descriptions of the context and numbers of scenarios and sessions, making it easier to draw conclusions about the effects of simulation.

## CONFLICT OF INTEREST

The authors declare no conflict of interest.

## AUTHOR CONTRIBUTIONS

All authors have agreed on the final version and meet at least one of the following criteria [recommended by the ICMJE (http://www.icmje.org/recommendations/)]: All author provided substantial contributions to conception and design, acquisition of data, analysis and interpretation of data, drafting the article or revising it critically for important intellectual content.

## Supporting information

Table S1

Table S2

Table S3

## Data Availability

The data used to support the findings of this study are available from the corresponding author on reasonable request.
