# Supplementary material for: The effect of multiple exposures in scenario‐based simulation—A mixed study systematic review
Source: Nurs Open. 2020 Sep 29;8(1):380–94. doi: 10.1002/nop2.639 (PMC7729777; doi:10.1002/nop2.639)
Supplement: Supplementary file 1 — Table S1 [file NOP2-8-380-s001.docx]

Supplemental Table 1 Example of search strategy, from CINAHL.

| **S1** | (MH "Patient Simulation") OR (MH "Vignettes") OR (MH "Computer Simulation") OR (MH "Simulations+") OR (MH "Simulations") OR simulated patient* OR “simulated patient” OR TI simulated patients OR AB simulated patients OR TI “simulated patients” OR scenario based simulation OR scenario-based simulation OR "clinical simulation" OR simulation activit* OR “simulation scenario" OR simulation scenario* OR "simulated scenario*" OR simulated scenario* OR "simulation education" OR simulated education OR simulation learning OR simulation-based scenario* OR “simulation-based scenario*” OR simulation-based education OR simulation-based activit* OR simulation based activit* OR healthcare simulation OR “healthcare simulation” OR simulation environment OR Simulation experience OR “Simulation experience” OR “Simulation experiences” | **Search modes** - Boolean/Phrase |
| --- | --- | --- |
| **S2** | "repeated exposure*"OR "multiple exposure*" OR repeated simulation OR repetition OR "simulation series" OR multiple simulation* OR "repeated scenario simulation" OR "repeated scenario" OR simulation sequence* OR simulation sequenc* OR repeated scenario* OR Multiple-simulation exposure OR Single-simulation exposure OR single simulation OR “single-simulation” OR single-simulation OR "simulation sequences" OR "simulation sequencing" OR "simulation sessions" OR "simulation session" OR scenario session OR "repeated sessions" OR "repeating sessions" OR "repeated simulation sessions" | **Search modes** - Boolean/Phrase |
| **S3** | (MH «Learning methods+») OR (MH Teaching methods+) OR “teaching strategies” OR (MH "Education+") OR (MH "Education, Nursing, Diploma Programs") OR (MH "Education, Nursing, Associate") | **Search modes** - Boolean/Phrase |
| **S4** | S1 AND S2 AND S3 |  |
