# Supplementary material for: The effect of multiple exposures in scenario‐based simulation—A mixed study systematic review
Source: Nurs Open. 2020 Sep 29;8(1):380–94. doi: 10.1002/nop2.639 (PMC7729777; doi:10.1002/nop2.639)
Supplement: Supplementary file 2 — Table S2 [file NOP2-8-380-s002.docx]

| Study  **Supplemental Table 2. Quality Appraisal of Selected Studies** | Aims and Objectives Clearly Stated | Study Design Adequately Described | Research Methods Appropriate | Adequate Description Sample, Inclusion/ Exclusion Criteria | Ethical Consideration Presented | Results Clear and Adequately Reported | Results Reported Related to Study Question and Literature | Limitations Presented | Implications Discussed | Study Sponsor |
| --- | --- | --- | --- | --- | --- | --- | --- | --- | --- | --- |
| Bussard (2018) | Yes | Yes | Yes | Yes | No | No | No | Yes | Yes | No |
| Chiang and Chan (2014) | Yes | Yes | Yes | Yes | Yes | Yes | Yes | Yes | Yes | Yes |
| Cummings and Connelly (2016) | No | Yes | Yes | Yes | Yes | Unclear | Yes | Yes | Unclear | No |
| Curl et al. (2016) | Yes | Yes | Yes | Yes | Yes* | Yes | No | No | Unclear | No |
| Díaz Agea et al. (2018) | Yes | Yes | Yes | Yes | Yes | Yes | Yes | Yes | Yes | No |
| Hansen and Bratt (2017) | Yes | Yes | Yes | Yes | Yes | Yes | Yes | Yes | Yes | No |
| Hart (2014) | Yes | Yes | Yes | Yes | Yes | Yes | Yes | Yes | Yes | No |
| Hicks (2009) | Yes | Yes | Yes | Yes | Yes | Yes | No | Yes | Unclear | No |
| Hill (2014) | Yes | Yes | Yes | Yes | Yes | No | No | Yes | Yes | No |
| Hoffmann et al. (2007) | Yes | Yes | Yes | Yes | Yes | Unclear | Yes | Yes | Unclear | No |
| Ironside et al. (2009) | Yes | Yes | Yes | Unclear | Yes* | Yes | Yes | Yes | Unclear | No |
| Lacue (2017) | Yes | Yes | Yes | Yes | Yes | Unclear | Unclear | Yes | Yes | No |
| Liaw et al. (2014) | Yes | Yes | Yes | Yes | Yes | Yes** | Yes | Yes | Yes | No |
| Mancini et al. (2019) | Yes | Yes | Yes | Unclear | No | Yes | Yes | Yes | Yes | No |
| Study | **Aims and Objectives Clearly Stated** | **Study Design Adequately Described** | **Research Methods Appropriate** | **Adequate Description Sample, Inclusion/ Exclusion Criteria** | **Ethical Consideration Presented** | **Results Clear and Adequately Reported** | **Results Reported Related to Study Question and Literature** | **Limitations Presented** | **Implications Discussed** | **Study Sponsor** |
| Melenovich (2012) | Yes | Yes | Yes | Yes | Yes | Yes | Yes | Yes | Yes | No |
| Meyer et al. (2011) | Yes | Yes | Yes | Yes | Yes | Yes | Yes | Yes | Yes | No |
| Mould et al. (2011) | Yes | Yes | Unclear | Unclear | Yes | Yes | Yes | Yes | Yes | No |
| Moule et al. (2008) | Yes | Yes | Yes | Yes | Yes | Unclear | Yes | Yes | Yes | No |
| Najjar 2015) | Yes | Yes | Unclear | Unclear | No | Yes | Unclear | Yes | Yes | No |
| Raman et al. (2019) | Yes | Yes | Yes | Unclear | Yes | Yes | Yes | Yes | Yes | No |
| Roh et al (2020) | Yes | Yes | Yes | Yes | Yes* | Yes | Yes | Yes | Yes | No |
| Schlairet and Pollock (2010) | Yes | Yes | Yes | Yes | Yes* | Yes | Yes | Yes | Yes | No |
| Schlairet (2012) | Yes | Yes | Yes | Yes | Yes* | Yes | Yes | Yes | Yes | No |
| Shin et al. (2015) | Yes | Yes | Yes | Yes | Yes | Yes | Yes | Yes | Yes | Yes*** |
| Thomas and Mackey (2012) | Yes | Yes | Yes | Yes | Yes | Unclear | Unclear | No | Yes | No |
| Unsworth et al. (2016) | Yes | Yes | Yes | Yes | Yes | Unclear | Yes | No | Unclear | No |
| Zapko et al. (2018) | Yes | Yes | Yes | Yes | Yes* | Yes | Yes | Yes | Yes | No |
