# Supplementary material for: The effect of multiple exposures in scenario‐based simulation—A mixed study systematic review
Source: Nurs Open. 2020 Sep 29;8(1):380–94. doi: 10.1002/nop2.639 (PMC7729777; doi:10.1002/nop2.639)
Supplement: Supplementary file 3 — Table S3 [file NOP2-8-380-s003.docx]

**Supplemental Table 3. Cochrane Risk of Bias assessment** (Díaz Agea et al. (2018) and Najjar et al. (2015) excluded because of their qualitative design.)

**Bussard (2018)**

| **Domain** | **Risk of bias**  *Low/ High/ Unclear* | **Support for judgement** | **Location in text** *(pg)* |
| --- | --- | --- | --- |
| Random sequence generation  *(selection bias)* | **High** | Not clearly stated in text. | 106 |
| Allocation concealment  *(selection bias)* | **High** | Not clearly stated in text. | 106 |
| Blinding of participants and personnel  *(performance bias)* | **Low** | The outcome is not likely to be influenced by lack of blinding. | 106 |
| Blinding of outcome assessment  *(detection bias)* | **Low** | The outcome measurement is not likely to be influenced by lack of blinding. | 106 |
| Incomplete outcome data  *(attrition bias)* | **High** | Not clearly stated in text. | 106 |
| Selective outcome reporting  *(reporting bias)* | **High** | Not clearly stated in text. Few findings presented | 106 |
| Other bias | **None** |  |  |

**Chiang and Chan (2014)**

| **Domain** | **Risk of bias**  *Low/ High/ Unclear* | **Support for judgement** | **Location in text** *(pg)* |
| --- | --- | --- | --- |
| Random sequence generation  *(selection bias)* | **High** | Not clearly stated in text. | 258, 259 |
| Allocation concealment  *(selection bias)* | **High** | Not clearly stated in text. | 258, 259 |
| Blinding of participants and personnel  *(performance bias)* | **Low** | The outcome is not likely to be influenced by lack of blinding. | 258, 259 |
| Blinding of outcome assessment  *(detection bias)* | **Low** | The outcome measurement is not likely to be influenced by lack of blinding. | 258, 259 |
| Incomplete outcome data  *(attrition bias)* | **Low** | Clearly stated in text. | 260 |
| Selective outcome reporting  *(reporting bias)* | **Low** | Clearly stated in text. | 260-262 |
| Other bias | **None** | - | - |

**Cummings and Connelly (2016)**

| **Domain** | **Risk of bias**  *Low/ High/ Unclear* | **Support for judgement** | **Location in text***(pg)* |
| --- | --- | --- | --- |
| Random sequence generation  *(selection bias)* | **High** | Not clearly stated in text. | 419 |
| Allocation concealment  *(selection bias)* | **High** | Not clearly stated in text. | 419 |
| Blinding of participants and personnel  *(performance bias)* | **Low** | The outcome is not likely to be influenced by lack of blinding. | 419 |
| Blinding of outcome assessment  *(detection bias)* | **Low** | The outcome measurement is not likely to be influenced by lack of blinding. | 419 |
| Incomplete outcome data  *(attrition bias)* | **Low** | Clearly stated in text. | 419, 420 |
| Selective outcome reporting  *(reporting bias)* | **Unclear** | Not clearly stated in text. | 420, 421 |
| Other bias | **None** | - | - |

**Curl et al. (2016)**

| **Domain** | **Risk of bias**  *Low/ High/ Unclear* | **Support for judgement** | **Location in text** *(pg)* |
| --- | --- | --- | --- |
| Random sequence generation  *(selection bias)* | **High** | Not clearly stated in text. | 73, 74 |
| Allocation concealment  *(selection bias)* | **High** | Not clearly stated in text. | 73, 74 |
| Blinding of participants and personnel  *(performance bias)* | **Low** | The outcome is not likely to be influenced by lack of blinding. | 73, 74 |
| Blinding of outcome assessment  *(detection bias)* | **Low** | The outcome measurement is not likely to be influenced by lack of blinding. | 73, 74 |
| Incomplete outcome data  *(attrition bias)* | **Low** | Clearly stated in text. | 76 |
| Selective outcome reporting  *(reporting bias)* | **Low** | Clearly stated in text. | 75,76 |
| Other bias | **None** | - | - |

**Hansen and Bratt (2017)**

| **Domain** | **Risk of bias**  *Low/ High/ Unclear* | **Support for judgement** | **Location in text** *(pg)* |
| --- | --- | --- | --- |
| Random sequence generation  *(selection bias)* | **Low** | Clearly stated in text. | 232 |
| Allocation concealment  *(selection bias)* | **Low** | Clearly stated in text. | 232 |
| Blinding of participants and personnel  *(performance bias)* | **Low** | The outcome is not likely to be influenced by lack of blinding. | 232 |
| Blinding of outcome assessment  *(detection bias)* | **Low** | The outcome measurement is not likely to be influenced by lack of blinding. | 232 |
| Incomplete outcome data  *(attrition bias)* | **Low** | Clearly stated in text. | 233 |
| Selective outcome reporting  *(reporting bias)* | **Low** | Clearly stated in text. | 233, 234 |
| Other bias | **None** | - | - |

**Hart et al. (2014)**

| **Domain** | **Risk of bias**  *Low/ High/ Unclear* | **Support for judgement** | **Location in text** *(pg)* |
| --- | --- | --- | --- |
| Random sequence generation  *(selection bias)* | **High** | Not clearly stated in text. | 27 |
| Allocation concealment  *(selection bias)* | **High** | Not clearly stated in text. | 27 |
| Blinding of participants and personnel  *(performance bias)* | **Low** | The outcome is not likely to be influenced by lack of blinding. | 27 |
| Blinding of outcome assessment  *(detection bias)* | **Low** | The outcome measurement is not likely to be influenced by lack of blinding. | 27 |
| Incomplete outcome data  *(attrition bias)* | **Low** | Clearly stated in text. | 29 |
| Selective outcome reporting  *(reporting bias)* | **Low** | Clearly stated in text. | 29,30 |
| Other bias | **None** | - | - |

**Hicks et al. (2009)**

| **Domain** | **Risk of bias**  *Low/ High/ Unclear* | **Support for judgement** | **Location in text** *(pg)* |
| --- | --- | --- | --- |
| Random sequence generation  *(selection bias)* | **Low** | Clearly stated in text. | 7 |
| Allocation concealment  *(selection bias)* | **Low** | Clearly stated in text. | 7 |
| Blinding of participants and personnel  *(performance bias)* | **Low** | The outcome is not likely to be influenced by lack of blinding. | 7, 16 |
| Blinding of outcome assessment  *(detection bias)* | **Low** | The outcome measurement is not likely to be influenced by lack of blinding. | 7, 16 |
| Incomplete outcome data  *(attrition bias)* | **Low** | Clearly stated in text. | 11 |
| Selective outcome reporting  *(reporting bias)* | **Low** | Clearly stated in text. | 11, 12, 13 |
| Other bias | **None** | - | - |

**Hill (2014)**

| **Domain** | **Risk of bias**  *Low/ High/Unclear* | **Support for judgement** | **Location in text**  *(pg)* |
| --- | --- | --- | --- |
| Random sequence generation  *(selection bias)* | **High** | Not clearly stated in text. | 24 |
| Allocation concealment  *(selection bias)* | **High** | Not clearly stated in text. | 24 |
| Blinding of participants and personnel  *(performance bias)* | **Low** | The outcome is not likely to be influenced by lack of blinding. | 24 |
| Blinding of outcome assessment  *(detection bias)* | **Low** | The outcome measurement is not likely to be influenced by lack of blinding. | 24 |
| Incomplete outcome data  *(attrition bias)* | **Low** | Clearly stated in text. | 31 |
| Selective outcome reporting  *(reporting bias)* | **High** | Not clearly stated in text. Few findings presented. | 31-33 |
| Other bias | **None** |  |  |

**Hoffmann et al. (2007)**

| **Domain** | **Risk of bias**  *Low/ High/ Unclear* | **Support for judgement** | **Location in text** *(pg)* |
| --- | --- | --- | --- |
| Random sequence generation  *(selection bias)* | **High** | Not clearly stated in text. | 111 |
| Allocation concealment  *(selection bias)* | **High** | Not clearly stated in text. | 111 |
| Blinding of participants and personnel  *(performance bias)* | **Low** | The outcome is not likely to be influenced by lack of blinding. | 111 |
| Blinding of outcome assessment  *(detection bias)* | **Low** | The outcome measurement is not likely to be influenced by lack of blinding. | 111 |
| Incomplete outcome data  *(attrition bias)* | **Low** | Clearly stated in text. | 113 |
| Selective outcome reporting  *(reporting bias)* | **High** | Not clearly stated in text. | 112 |
| Other bias | **None** | - | - |

**Ironside et al. (2009)**

| **Domain** | **Risk of bias**  *Low/ High/Unclear* | **Support for judgement** | **Location in text**  *(pg)* |
| --- | --- | --- | --- |
| Random sequence generation  *(selection bias)* | **High** | Not clearly stated in text. | 334 |
| Allocation concealment  *(selection bias)* | **High** | Not clearly stated in text. | 334 |
| Blinding of participants and personnel  *(performance bias)* | **Low** | The outcome is not likely to be influenced by lack of blinding. | 334 |
| Blinding of outcome assessment  *(detection bias)* | **Low** | The outcome measurement is not likely to be influenced by lack of blinding. | 334 |
| Incomplete outcome data  *(attrition bias)* | **High** | Not clearly stated in text. | 336 |
| Selective outcome reporting  *(reporting bias)* | **Unclear** | Not clearly stated in text. | 336 |
| Other bias | **None** | - | - |

**Lacue (2017)**

| **Domain** | **Risk of bias**  *Low/ High/ Unclear* | **Support for judgement** | **Location in text** *(pg)* |
| --- | --- | --- | --- |
| Random sequence generation  *(selection bias)* | **High** | Not clearly stated in text. | 19, 20 |
| Allocation concealment  *(selection bias)* | **High** | Not clearly stated in text. | 19, 20 |
| Blinding of participants and personnel  *(performance bias)* | **Low** | The outcome is not likely to be influenced by lack of blinding. | 19, 20 |
| Blinding of outcome assessment  *(detection bias)* | **Low** | The outcome measurement is not likely to be influenced by lack of blinding. | 19, 20 |
| Incomplete outcome data  *(attrition bias)* | **Low** | Clearly stated in text. | 29 |
| Selective outcome reporting  *(reporting bias)* | **High** | Not clearly stated in text. | 29-38 |
| Other bias | **None** | - | - |

**Liew et al. (2014)**

| **Domain** | **Risk of bias**  *Low/ High/ Unclear* | **Support for judgement** | **Location in text** *(pg)* |
| --- | --- | --- | --- |
| Random sequence generation  *(selection bias)* | **High** | Not clearly stated in text. | 352 |
| Allocation concealment  *(selection bias)* | **High** | Not clearly stated in text. | 352 |
| Blinding of participants and personnel  *(performance bias)* | **Low** | The outcome is not likely to be influenced by lack of blinding. | 352 |
| Blinding of outcome assessment  *(detection bias)* | **Low** | The outcome measurement is not likely to be influenced by lack of blinding. | 352 |
| Incomplete outcome data  *(attrition bias)* | **Low** | Clearly stated in text. | 352 |
| Selective outcome reporting  *(reporting bias)* | **Low** | Clearly stated in text. | 352 |
| Other bias | **None** | - | - |

**Mancini et al. (2019)**

| **Domain** | **Risk of bias**  *Low/ High/Unclear* | **Support for judgement** | **Location in text**  *(pg)* |
| --- | --- | --- | --- |
| Random sequence generation  *(selection bias)* | **High** | Clearly stated in text. | 563, 565 |
| Allocation concealment  *(selection bias)* | **High** | Clearly stated in text. | 563, 565 |
| Blinding of participants and personnel  *(performance bias)* | **Low** | The outcome is not likely to be influenced by lack of blinding. |  |
| Blinding of outcome assessment  *(detection bias)* | **Low** | The outcome measurement is not likely to be influenced by lack of blinding. |  |
| Incomplete outcome data  *(attrition bias)* | **Low** | Clearly stated in text. | 563 |
| Selective outcome reporting  *(reporting bias)* | **Low** | Clearly stated in text. | 565-566 |
| Other bias | **None** |  |  |

**Melenovich (2012)**

| **Domain** | **Risk of bias**  *Low/ High/ Unclear* | **Support for judgement** | **Location in text** *(pg)* |
| --- | --- | --- | --- |
| Random sequence generation  *(selection bias)* | **Low** | Clearly stated in text. | 76, 77 |
| Allocation concealment  *(selection bias)* | **Low** | Clearly stated in text. | 76, 77 |
| Blinding of participants and personnel  *(performance bias)* | **Low** | The outcome is not likely to be influenced by lack of blinding. | 76, 77 |
| Blinding of outcome assessment  *(detection bias)* | **Low** | The outcome measurement is not likely to be influenced by lack of blinding. | 76, 77 |
| Incomplete outcome data  *(attrition bias)* | **Low** | Clearly stated in text. | 79, 83 |
| Selective outcome reporting  *(reporting bias)* | **Low** | Clearly stated in text. | 82-117 |
| Other bias | **Small sample size** | Clearly stated in text. | 83 |

**Meyer et al. (2011)**

| **Domain** | **Risk of bias**  *Low/ High/ Unclear* | **Support for judgement** | **Location in text** *(pg)* |
| --- | --- | --- | --- |
| Random sequence generation  *(selection bias)* | **Low** | Clearly stated in text. | 270 |
| Allocation concealment  *(selection bias)* | **Low** | Clearly stated in text. | 270 |
| Blinding of participants and personnel  *(performance bias)* | **Low** | The outcome is not likely to be influenced by lack of blinding. | 275 |
| Blinding of outcome assessment  *(detection bias)* | **Low** | The outcome measurement is not likely to be influenced by lack of blinding. | 275 |
| Incomplete outcome data  *(attrition bias)* | **Low** | Clearly stated in text. | 272 |
| Selective outcome reporting  *(reporting bias)* | **Low** | Clearly stated in text. | 272, 273 |
| Other bias | **None** | . | - |

**Mould et al. (2011)**

| **Domain** | **Risk of bias**  *Low/ High/ Unclear* | **Support for judgement** | **Location in text** *(pg)* |
| --- | --- | --- | --- |
| Random sequence generation  *(selection bias)* | **High** | Not clearly stated in text. | 183 |
| Allocation concealment  *(selection bias)* | **High** | Not clearly stated in text. | 183 |
| Blinding of participants and personnel  *(performance bias)* | **Low** | The outcome is not likely to be influenced by lack of blinding. | 183 |
| Blinding of outcome assessment  *(detection bias)* | **Low** | The outcome measurement is not likely to be influenced by lack of blinding. | 183 |
| Incomplete outcome data  *(attrition bias)* | **Low** | Not clearly stated in text. | 184 |
| Selective outcome reporting  *(reporting bias)* | **Low** | Clearly stated in text. | 184-186 |
| Other bias | **None** |  | - |

**Moule et al. (2008)**

| **Domain** | **Risk of bias**  *Low/ High/ Unclear* | **Support for judgement** | **Location in text** *(pg)* |
| --- | --- | --- | --- |
| Random sequence generation  *(selection bias)* | **High** | Not clearly stated in text. | 791 |
| Allocation concealment  *(selection bias)* | **High** | Not clearly stated in text. | 791 |
| Blinding of participants and personnel  *(performance bias)* | **Low** | The outcome is not likely to be influenced by lack of blinding. | 791 |
| Blinding of outcome assessment  *(detection bias)* | **Low** | The outcome measurement is not likely to be influenced by lack of blinding. | 791 |
| Incomplete outcome data  *(attrition bias)* | **Low** | Clearly stated in text. | 793 |
| Selective outcome reporting  *(reporting bias)* | **Unclear** | Not clearly stated in text. | 793-795 |
| Other bias | **Small sample size** | Clearly stated in text. | 791 |

| **Domain** | **Risk of bias**  *Low/ High/Unclear* | **Support for judgement** | **Location in text**  *(pg)* |
| --- | --- | --- | --- |
| Random sequence generation  *(selection bias)* | **High** | Clearly stated in text. | 435 |
| Allocation concealment  *(selection bias)* | **High** | Clearly stated in text. | 435 |
| Blinding of participants and personnel  *(performance bias)* | **Low** | The outcome is not likely to be influenced by lack of blinding. |  |
| Blinding of outcome assessment  *(detection bias)* | **Low** | The outcome measurement is not likely to be influenced by lack of blinding. |  |
| Incomplete outcome data  *(attrition bias)* | **Low** | Clearly stated in text. | 436 |
| Selective outcome reporting  *(reporting bias)* | **Low** | Clearly stated in text. | 437-438 |
| Other bias | **None** |  |  |

**Raman et al. (2019)**

**Roh et al. (2020)**

| **Domain** | **Risk of bias**  *Low/ High/Unclear* | **Support for judgement** | **Location in text**  *(pg)* |
| --- | --- | --- | --- |
| Random sequence generation  *(selection bias)* | **High** | Clearly stated in text. | 2 |
| Allocation concealment  *(selection bias)* | **High** | Clearly stated in text. | 2 |
| Blinding of participants and personnel  *(performance bias)* | **Low** | The outcome is not likely to be influenced by lack of blinding. |  |
| Blinding of outcome assessment  *(detection bias)* | **Low** | The outcome measurement is not likely to be influenced by lack of blinding. |  |
| Incomplete outcome data  *(attrition bias)* | **Low** | Clearly stated in text. | 3 |
| Selective outcome reporting  *(reporting bias)* | **Low** | Clearly stated in text. | 4-5 |
| Other bias | **None** |  |  |

**Schlairet and Pollock (2010)**

| **Domain** | **Risk of bias**  *Low/ High/ Unclear* | **Support for judgement** | **Location in text** *(pg)* |
| --- | --- | --- | --- |
| Random sequence generation  *(selection bias)* | **Low** | Clearly stated in text. | 44 |
| Allocation concealment  *(selection bias)* | **Low** | Clearly stated in text. | 44 |
| Blinding of participants and personnel  *(performance bias)* | **Low** | The outcome is not likely to be influenced by lack of blinding. | 44 |
| Blinding of outcome assessment  *(detection bias)* | **Low** | The outcome measurement is not likely to be influenced by lack of blinding. | 44 |
| Incomplete outcome data  *(attrition bias)* | **Low** | Clearly stated in text. | 44 |
| Selective outcome reporting  *(reporting bias)* | **Low** | Clearly stated in text. | 44, 45 |
| Other bias | **Small sample size** | Clearly stated in text. | 45 |

**Schlairet and Fenster (2012)**

| **Domain** | **Risk of bias**  *Low/ High/ Unclear* | **Support for judgement** | **Location in text** *(pg)* |
| --- | --- | --- | --- |
| Random sequence generation  *(selection bias)* | **Low** | Clearly stated in text. | 669 |
| Allocation concealment  *(selection bias)* | **Low** | Clearly stated in text. | 669 |
| Blinding of participants and personnel  *(performance bias)* | **Low** | The outcome is not likely to be influenced by lack of blinding. | 669, 670 |
| Blinding of outcome assessment  *(detection bias)* | **Low** | The outcome measurement is not likely to be influenced by lack of blinding. | 669. 670 |
| Incomplete outcome data  *(attrition bias)* | **Low** | Clearly stated in text. | 671 |
| Selective outcome reporting  *(reporting bias)* | **Low** | Clearly stated in text. | 671-673 |
| Other bias | **Small sample size** | - | - |

**Shin et al. (2015)**

| **Domain** | **Risk of bias**  *Low/ High/ Unclear* | **Support for judgement** | **Location in text** *(pg)* |
| --- | --- | --- | --- |
| Random sequence generation  *(selection bias)* | **High** | Not clearly stated in text. | 539 |
| Allocation concealment  *(selection bias)* | **High** | Not clearly stated in text. | 539 |
| Blinding of participants and personnel  *(performance bias)* | **Low** | The outcome is not likely to be influenced by lack of blinding. | 538 |
| Blinding of outcome assessment  *(detection bias)* | **Low** | The outcome measurement is not likely to be influenced by lack of blinding. | 538 |
| Incomplete outcome data  *(attrition bias)* | **Low** | Clearly stated in text. | 538 |
| Selective outcome reporting  *(reporting bias)* | **Low** | Clearly stated in text. | 539, 540 |
| Other bias | **None** | - | - |

**Thomas and Mackey (2012)**

| **Domain** | **Risk of bias**  *Low/ High/ Unclear* | **Support for judgement** | **Location in text** *(pg)* |
| --- | --- | --- | --- |
| Random sequence generation  *(selection bias)* | **High** | Not clearly stated in text. | 237 |
| Allocation concealment  *(selection bias)* | **High** | Not clearly stated in text. | 237 |
| Blinding of participants and personnel  *(performance bias)* | **Low** | The outcome is not likely to be influenced by lack of blinding. | 237 |
| Blinding of outcome assessment  *(detection bias)* | **Low** | The outcome measurement is not likely to be influenced by lack of blinding. | 237 |
| Incomplete outcome data  *(attrition bias)* | **Unclear** | Not clearly stated in text. | 237 |
| Selective outcome reporting  *(reporting bias)* | **Low** | Clearly stated in text. | 237, 238 |
| Other bias | **Small sample size** | Clearly stated in text. | 237 |

**Unsworth et al. (2016)**

| **Domain** | **Risk of bias**  *Low/ High/ Unclear* | **Support for judgement** | **Location in text** *(pg)* |
| --- | --- | --- | --- |
| Random sequence generation  *(selection bias)* | **High** | Not clearly stated in text. | 48 |
| Allocation concealment  *(selection bias)* | **High** | Not clearly stated in text. | 48 |
| Blinding of participants and personnel  *(performance bias)* | **Low** | The outcome is not likely to be influenced by lack of blinding. | 49 |
| Blinding of outcome assessment  *(detection bias)* | **Low** | The outcome measurement is not likely to be influenced by lack of blinding. | 49 |
| Incomplete outcome data  *(attrition bias)* | **Low** | Clearly stated in text. | 49 |
| Selective outcome reporting  *(reporting bias)* | **Low** | Clearly stated in text. | 50-52 |
| Other bias | **None** | - | - |

**Zapko et al. (2018)**

| **Domain** | **Risk of bias**  *Low/ High/ Unclear* | **Support for judgement** | **Location in text** *(pg)* |
| --- | --- | --- | --- |
| Random sequence generation  *(selection bias)* | **High** | Not clearly stated in text. | 29 |
| Allocation concealment  *(selection bias)* | **High** | Not clearly stated in text. | 29 |
| Blinding of participants and personnel  *(performance bias)* | **Low** | The outcome is not likely to be influenced by lack of blinding. | 29 |
| Blinding of outcome assessment  *(detection bias)* | **Low** | The outcome measurement is not likely to be influenced by lack of blinding. | 29 |
| Incomplete outcome data  *(attrition bias)* | **Low** | Clearly stated in text. | 29 |
| Selective outcome reporting  *(reporting bias)* | **Low** | Clearly stated in text. | 30-33 |
| Other bias | **None** | - | - |
